# Supplementary figures and images for: Diversity and Reassortment Rate of Influenza A Viruses in Wild Ducks and Gulls
Source: Viruses. 2021 May 27;13(6):1010. doi: 10.3390/v13061010 (PMC8230314; doi:10.3390/v13061010)

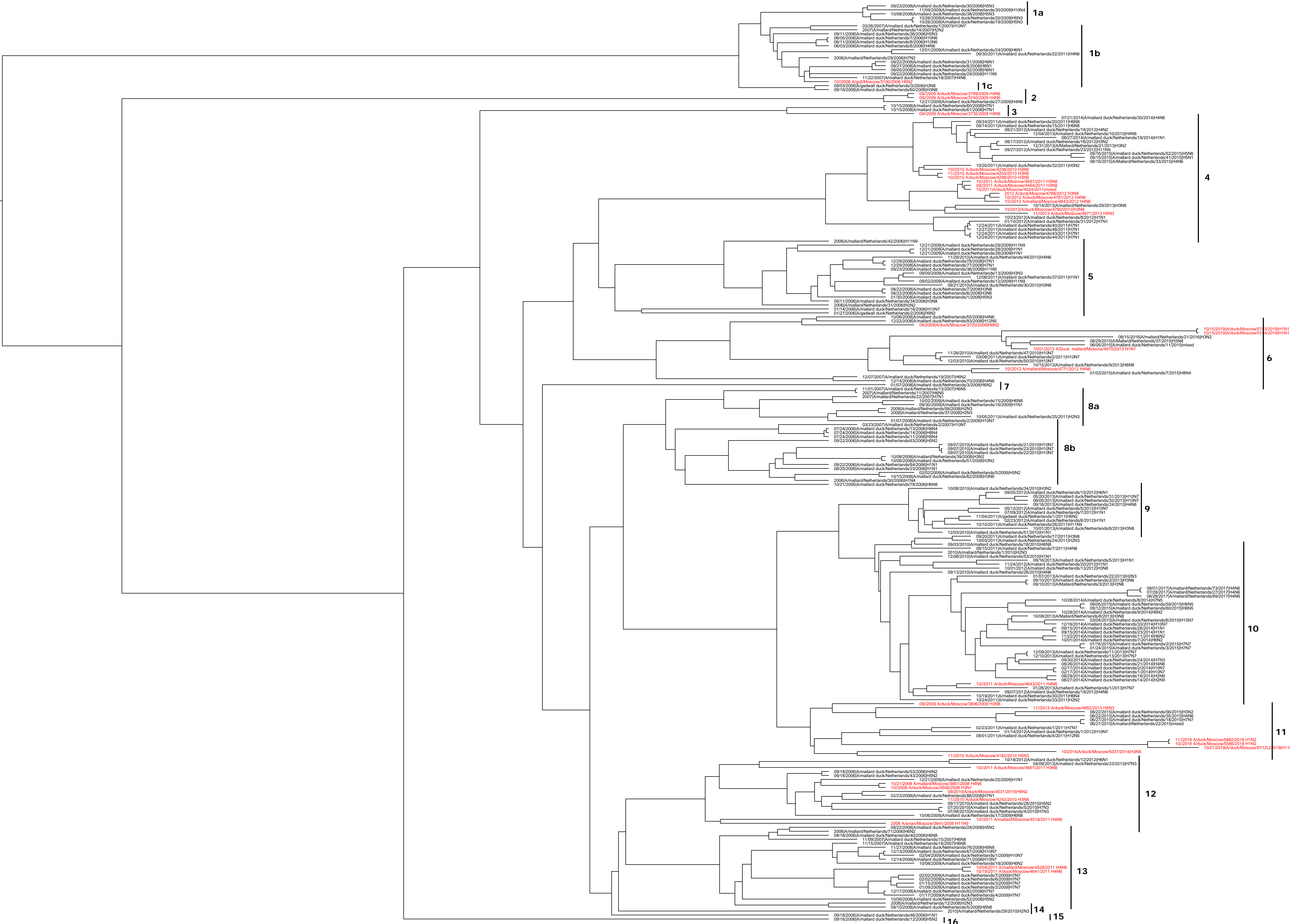

Supplement: Supplementary file 1 [file viruses-13-01010-s001.zip › Figure S1 PB2 duck.pdf]

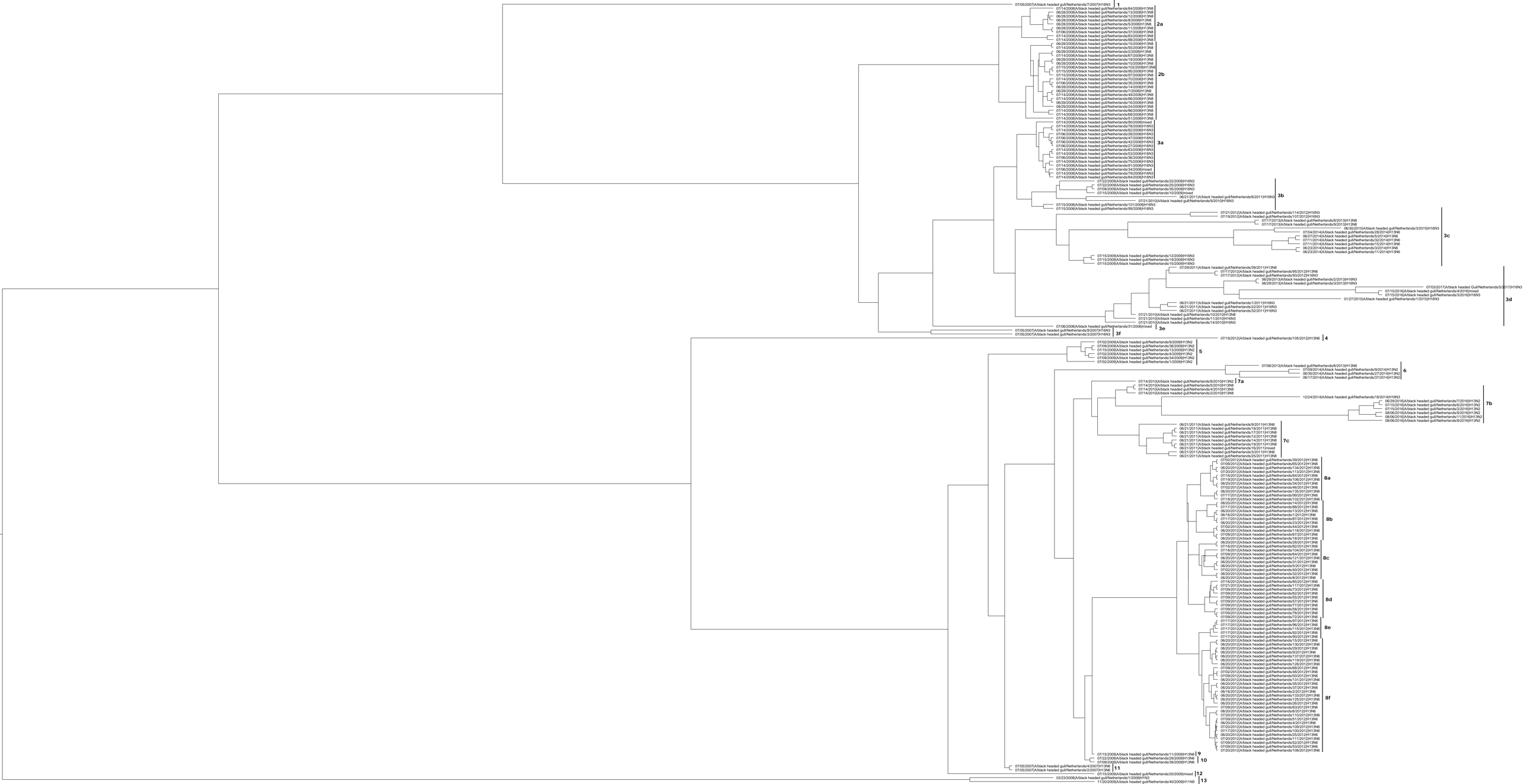

Supplement: Supplementary file 1 [file viruses-13-01010-s001.zip › Figure S11 MP gull.pdf]

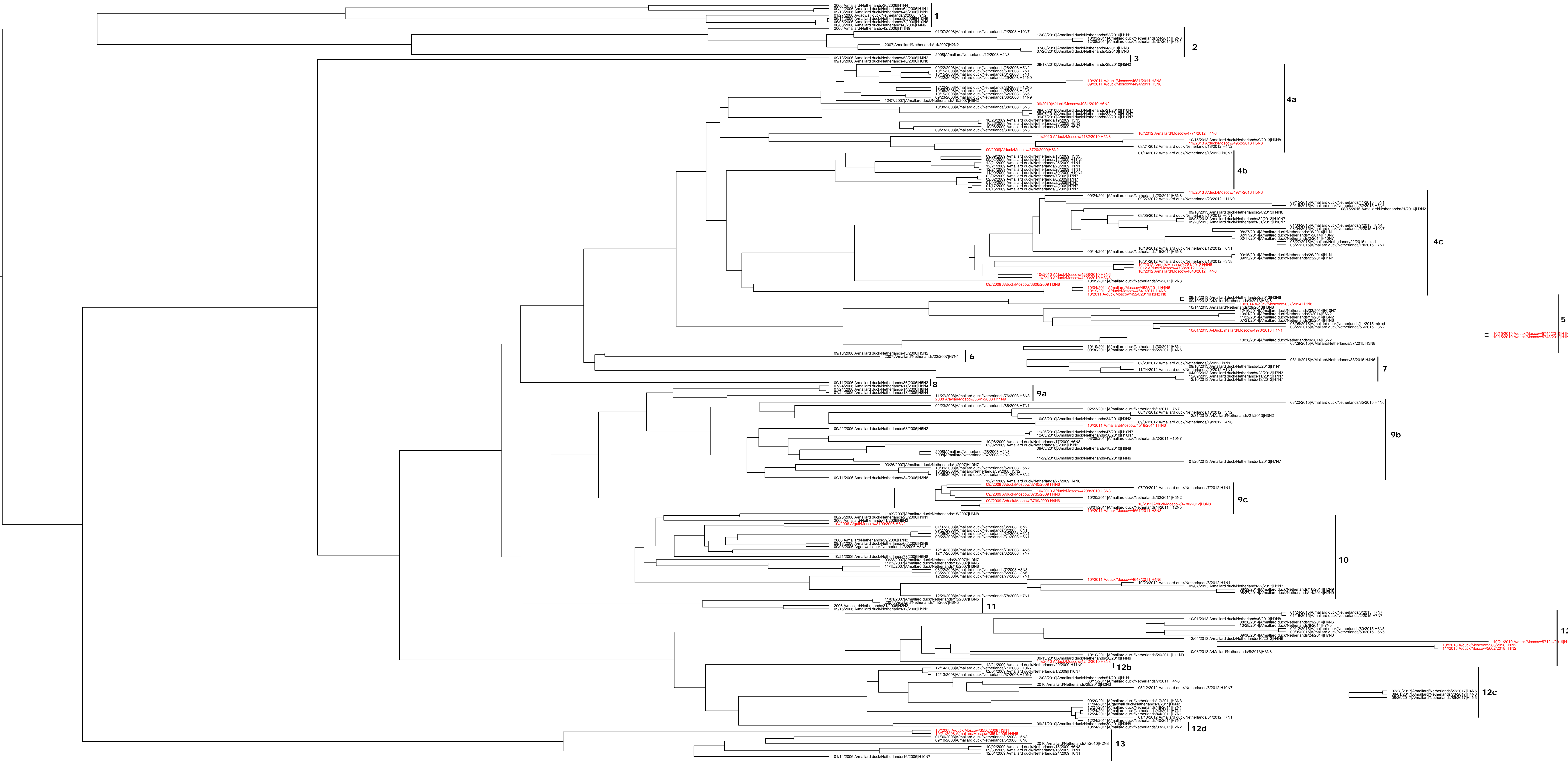

Supplement: Supplementary file 1 [file viruses-13-01010-s001.zip › Figure S2 PB1 duck.pdf]

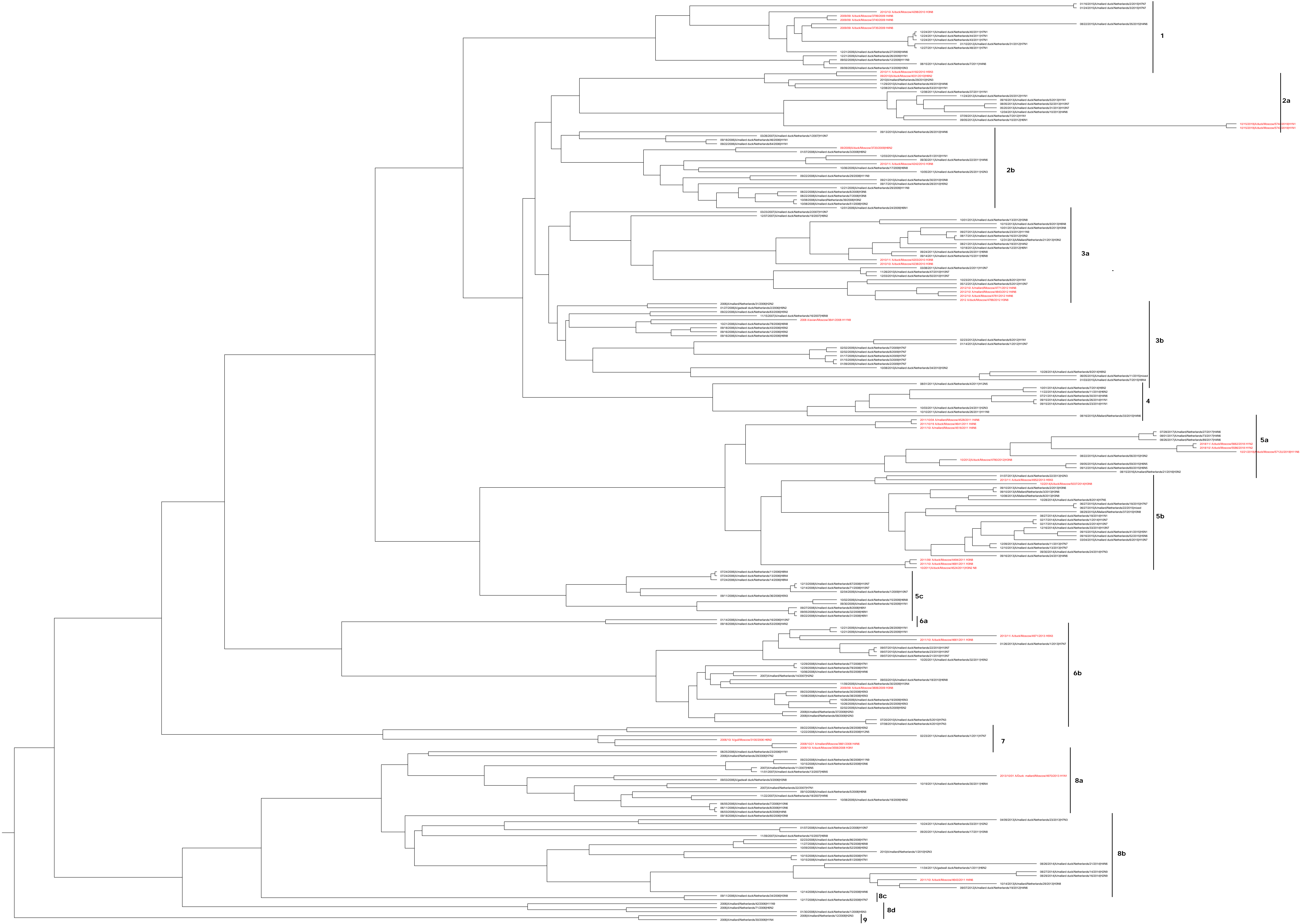

Supplement: Supplementary file 1 [file viruses-13-01010-s001.zip › Figure S3 PA duck.pdf]

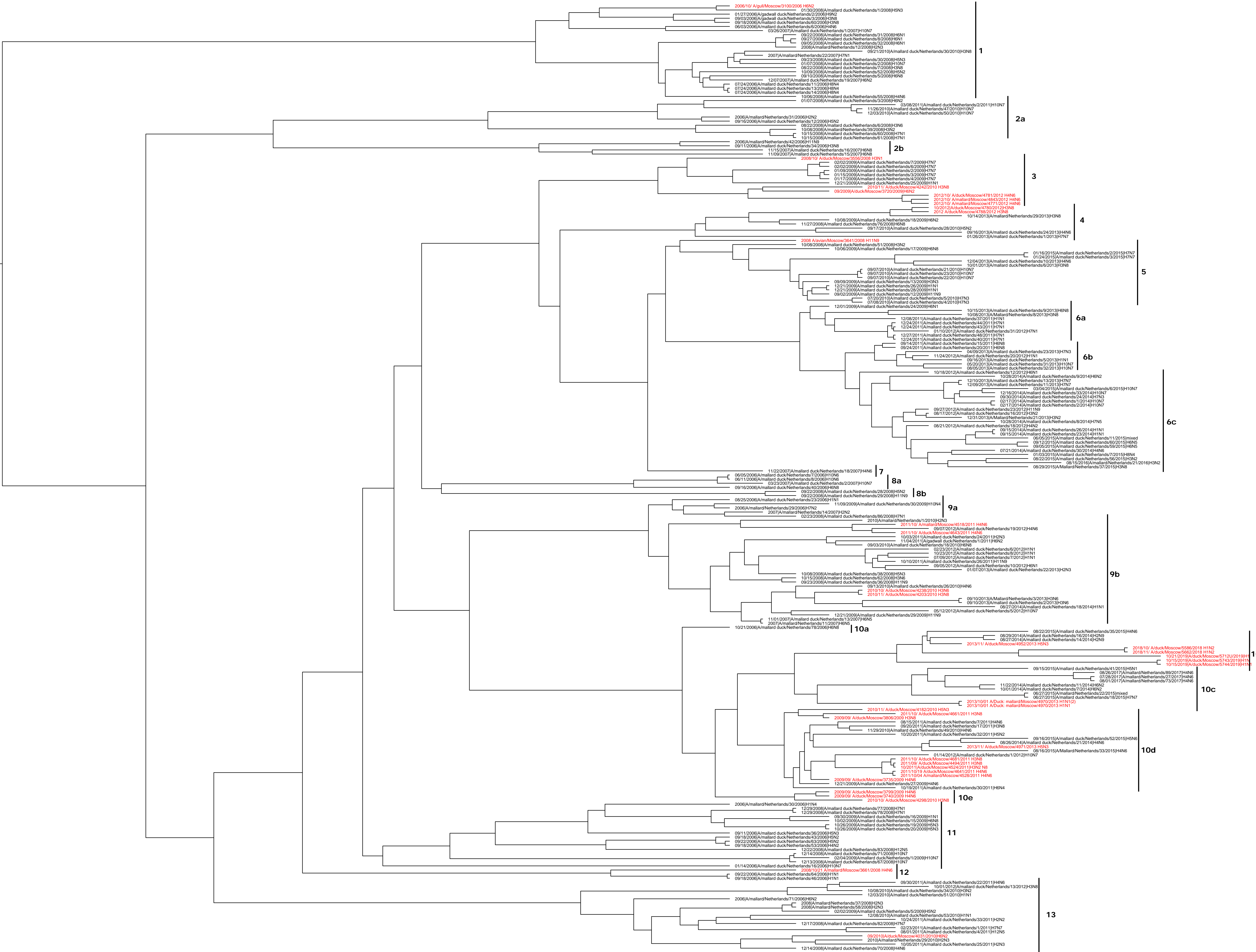

Supplement: Supplementary file 1 [file viruses-13-01010-s001.zip › Figure S4 NP duck.pdf]

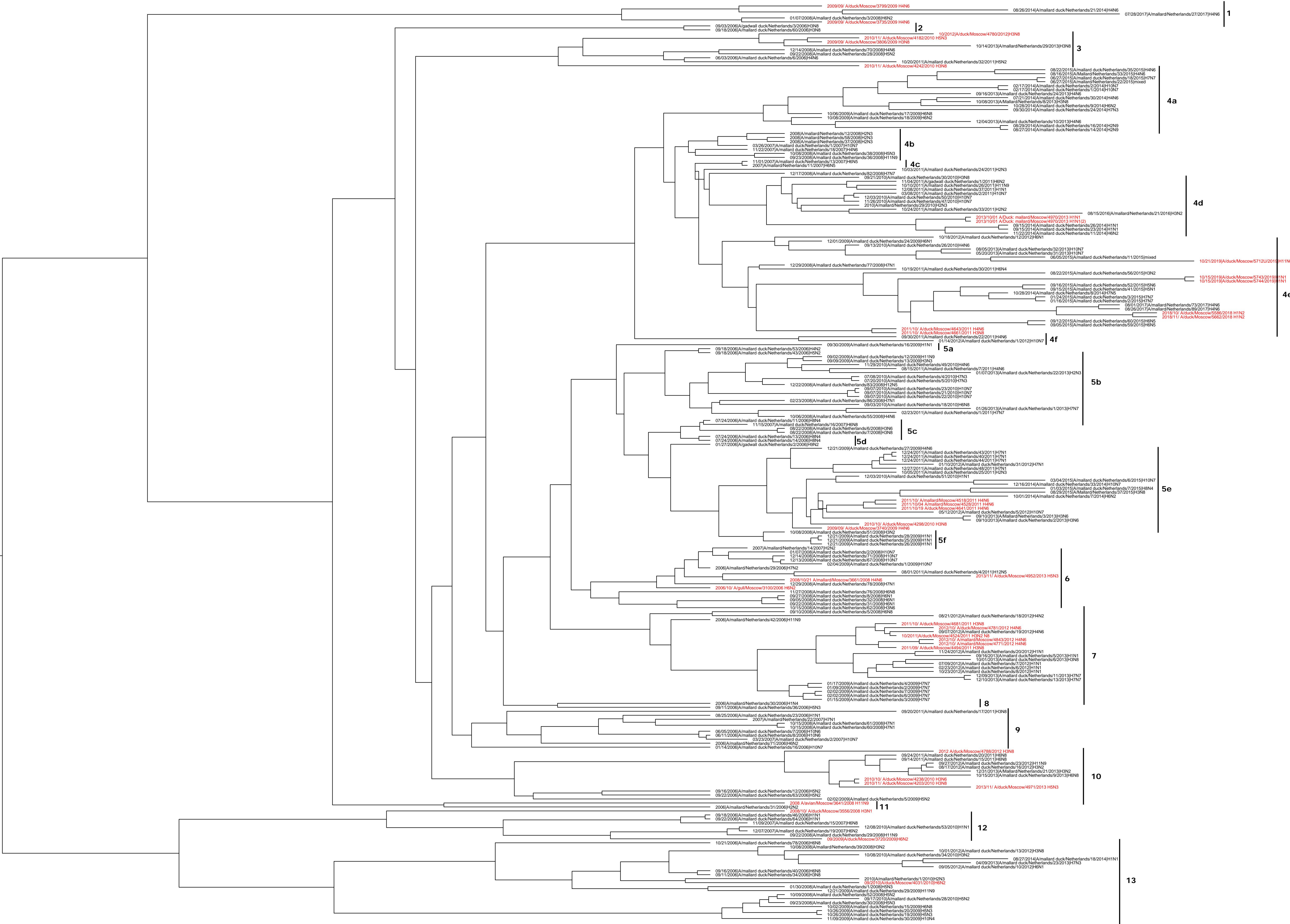

Supplement: Supplementary file 1 [file viruses-13-01010-s001.zip › Figure S5 MP duck.pdf]

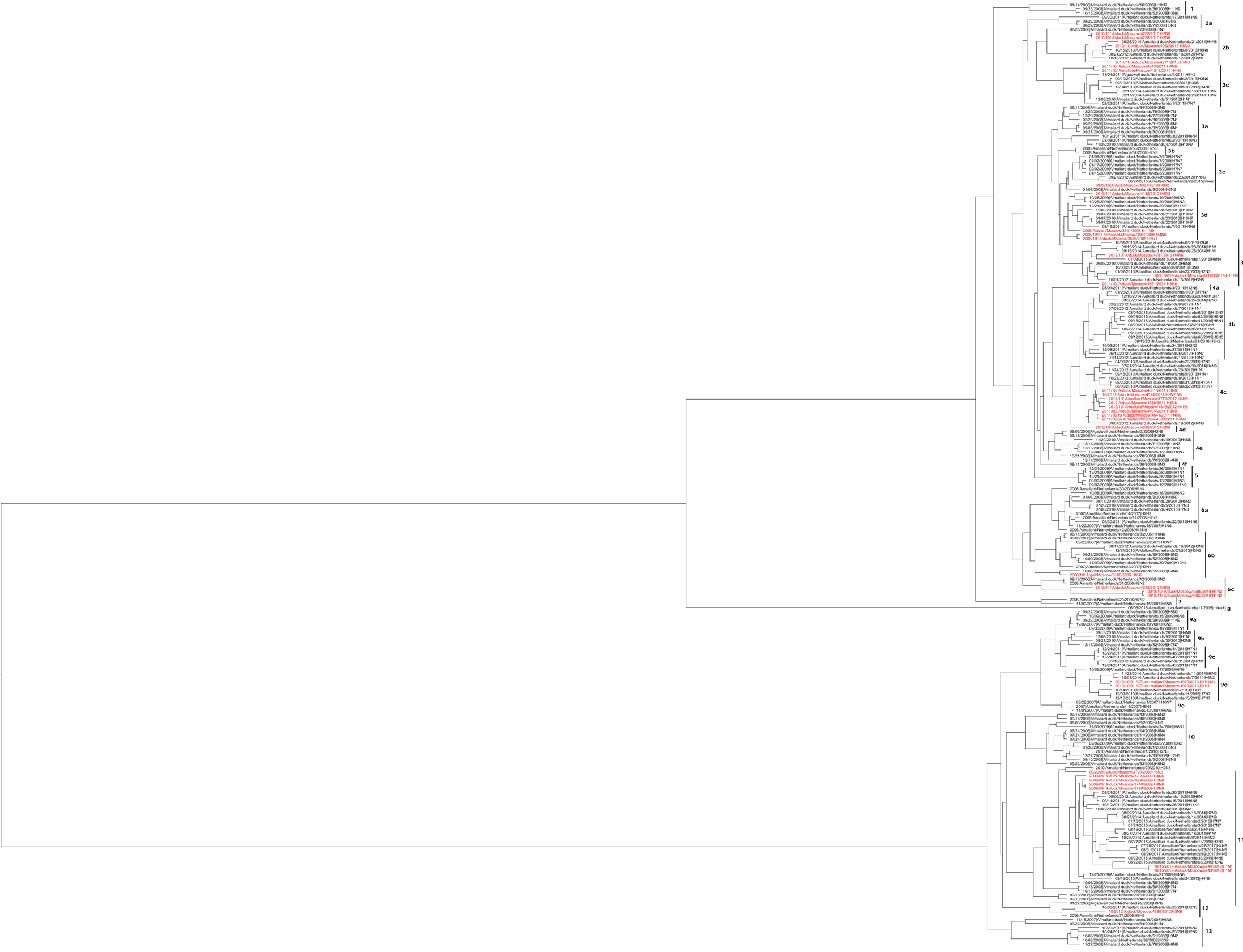

Supplement: Supplementary file 1 [file viruses-13-01010-s001.zip › Figure S6 NS duck.pdf]

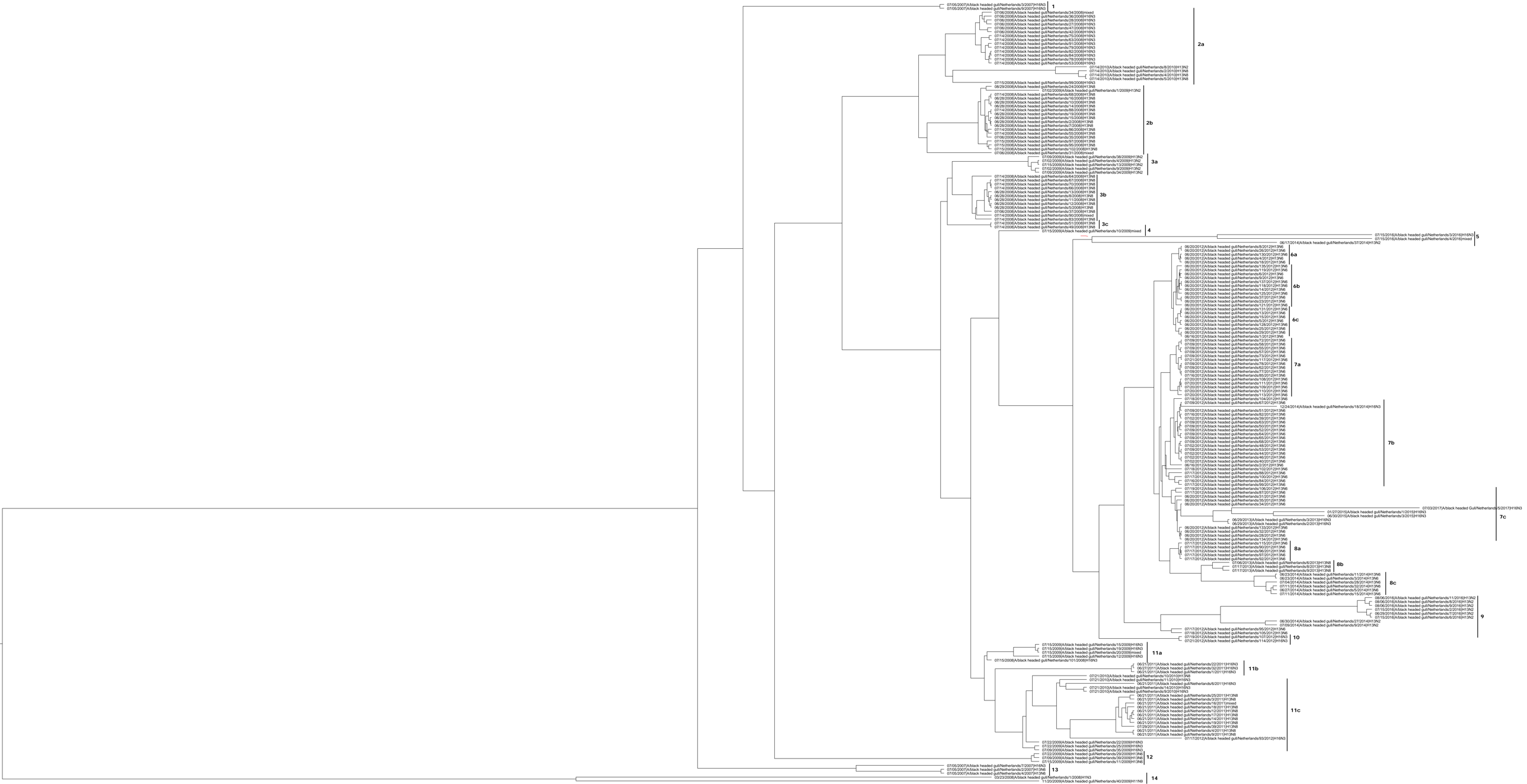

Supplement: Supplementary file 1 [file viruses-13-01010-s001.zip › Figure S7 PB2 gull.pdf]
