# Supplementary material for: Diversity and Reassortment Rate of Influenza A Viruses in Wild Ducks and Gulls
Source: Viruses. 2021 May 27;13(6):1010. doi: 10.3390/v13061010 (PMC8230314; doi:10.3390/v13061010)
Supplement: Supplementary file 1 [file Figure S10 NP gull.pdf › Figure S10 NP gull.pdf]

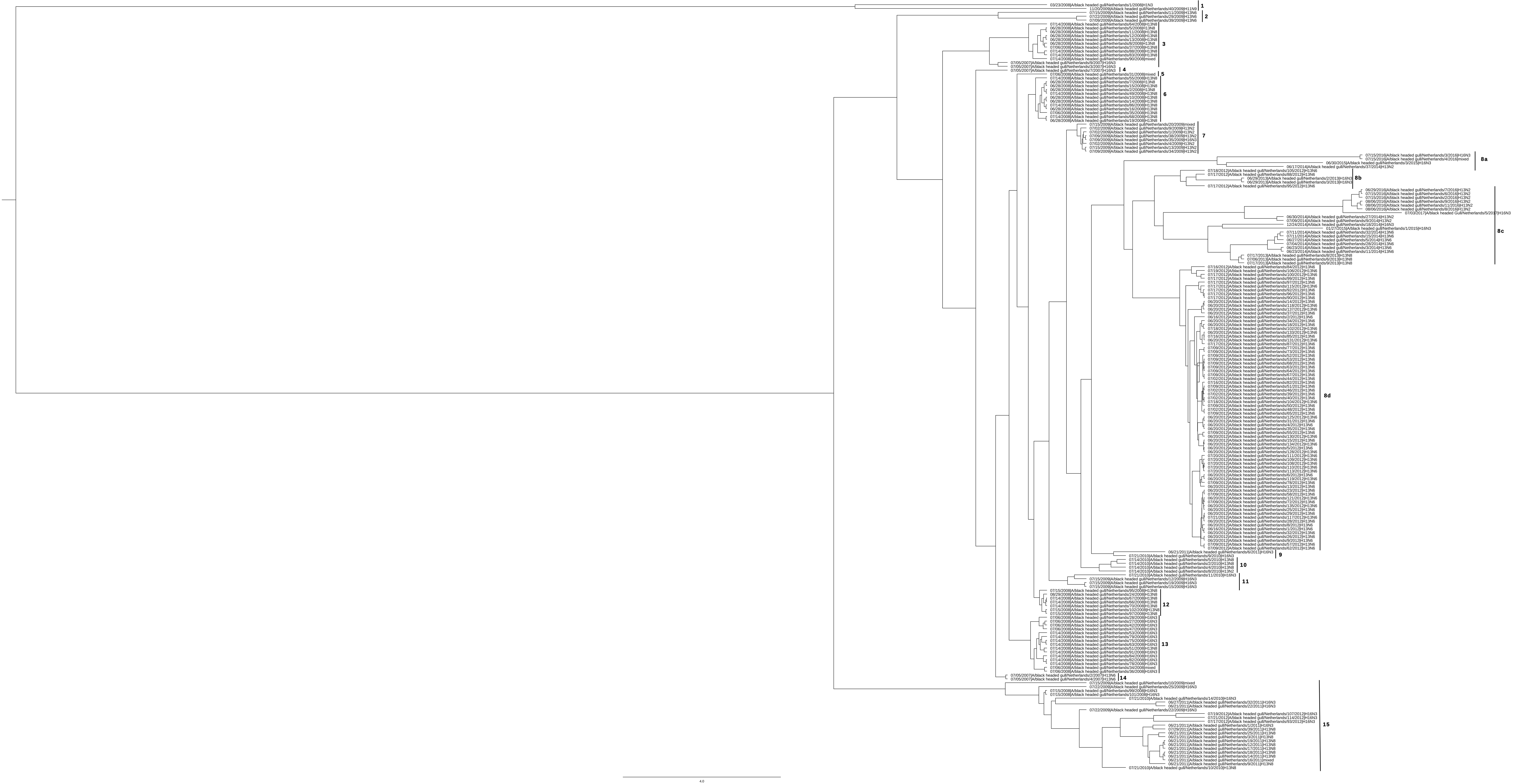
1
2
3
4
5
6
7
8a
8b
8c
8d
9
10
11
12
13
14
15
4.0
