# Supplementary material for: Diversity and Reassortment Rate of Influenza A Viruses in Wild Ducks and Gulls
Source: Viruses. 2021 May 27;13(6):1010. doi: 10.3390/v13061010 (PMC8230314; doi:10.3390/v13061010)
Supplement: Supplementary file 1 [file Figure S12 NS gull.pdf › Figure S12 NS gull.pdf]

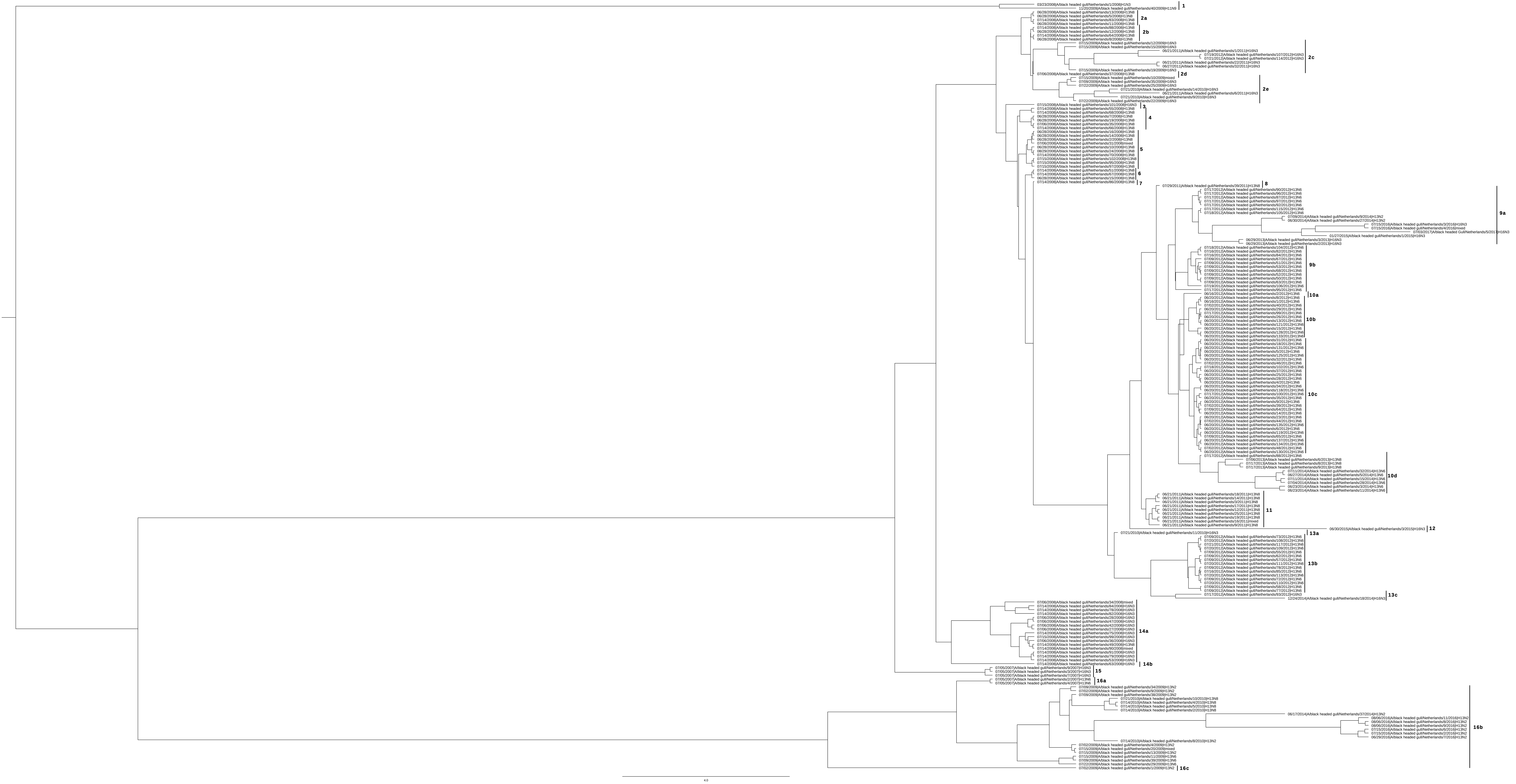

1
2a
2b
2c
2d
2e
3
4
5
6
7
8
9a
9b
10a
10b
10c
10d
11
12
13a
13b
13c
14a
14b
15
16a
16b
16c
4.0
