# Supplementary material for: Diversity and Reassortment Rate of Influenza A Viruses in Wild Ducks and Gulls
Source: Viruses. 2021 May 27;13(6):1010. doi: 10.3390/v13061010 (PMC8230314; doi:10.3390/v13061010)
Supplement: Supplementary file 1 [file Figure S8 PB1 gull.pdf › Figure S8 PB1 gull.pdf]

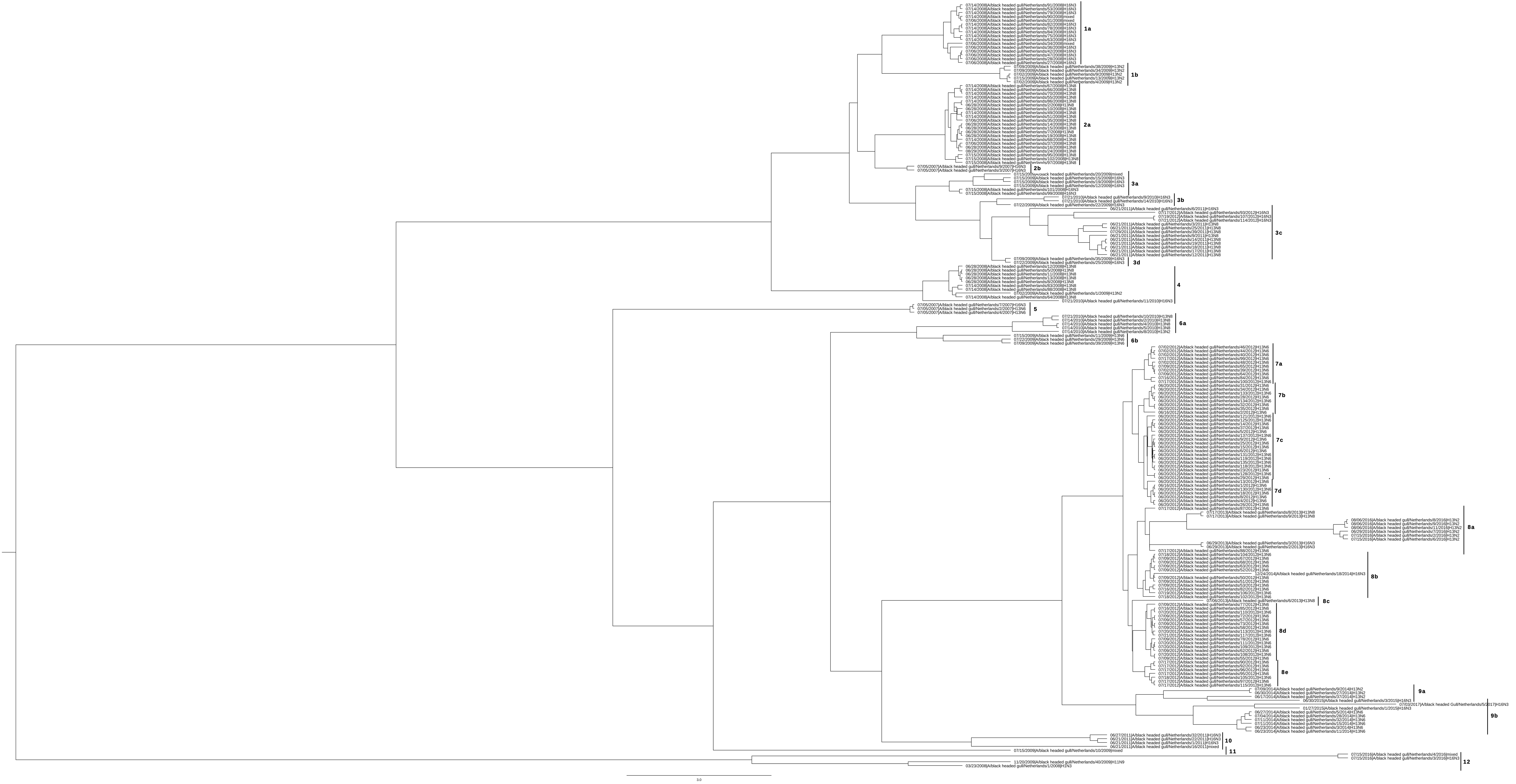
1a
1b
2a
2b
3a
3b
3c
3d
4
5
6a
6b
7a
7b
7c
7d
8a
8b
8c
8d
8e
9a
9b
10
11
12
3.0
