# Supplementary material for: Diversity and Reassortment Rate of Influenza A Viruses in Wild Ducks and Gulls
Source: Viruses. 2021 May 27;13(6):1010. doi: 10.3390/v13061010 (PMC8230314; doi:10.3390/v13061010)
Supplement: Supplementary file 1 [file Figure S9 PA gull.pdf › Figure S9 PA gull.pdf]

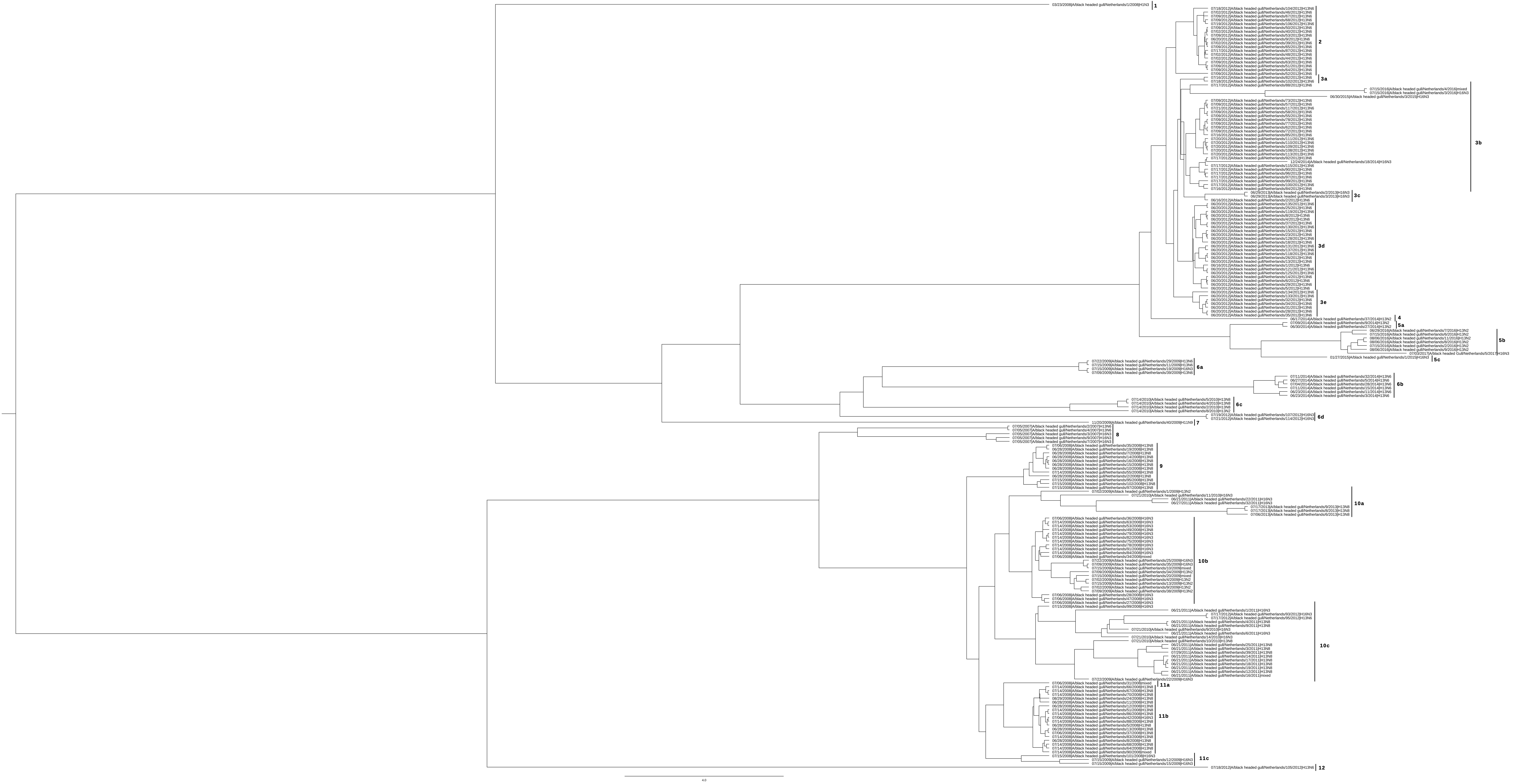
1
2
3a
3b
3c
3d
3e
4
5a
5b
5c
6a
6b
6c
6d
7
8
9
10a
10b
10c
11a
11b
11c
12
4.0
